# The Relationship between Exposure to Lead-Containing Welding Fumes and the Levels of Reproductive Hormones

**DOI:** 10.5334/aogh.2617

**Published:** 2019-10-15

**Authors:** Somayeh Farhang Dehghan, Younes Mehrifar, Arash Ardalan

**Affiliations:** 1 Department of Occupational Health and Safety at Work, Workplace Health Promotion Research Center, School of Public Health and Safety, Shahid Beheshti University of Medical Sciences, Tehran, IR; 2 Department of Occupational Health Engineering, Student Research Committee, School of Public Health, Isfahan University of Medical Sciences, Isfahan, IR; 3 Department of Cancer Database Management, St’ Joseph Hospital, Burbank, CA, US

## Abstract

**Background:**

Inhalation of lead oxide fumes may cause lead poisoning. Lead has been proven to have harmful effects on different organs. This study aimed to investigate the relationship between the concentration of lead fumes and the levels of reproductive hormones among exposed welders.

**Methods:**

A total number of 165 individuals of a construction industry of water pipelines, including 85 welders as the exposure group and 80 administrative staff as the non-exposure group were selected for study. The National Institute for Occupational Safety and Health (NIOSH) 7300 method was used for the purpose of sampling and analysis of lead fumes. Likewise, the NIOSH 8003 method was employed to determine the blood lead level. The level of luteinizing hormone (LH), follicle-stimulating hormone (FSH), and testosterone were measured by Chemiluminescence immunoassay(CLIA) test. Data analyses were done by SPSS ver.21 using descriptive statistics, Student›s t-test and Spearman›s correlation test.

**Results:**

The average concentration of lead fumes in the breathing zone and blood were 0.57 ± 0.12 mg/m^3^ and 460.28 ± 93.65 μg/L, correspondingly, which both were significantly higher than threshold limit values (TLV) and biological exposure index (BEI) recommended by American Conference of Governmental Industrial Hygienists (ACGIH)(P < 0.05). The mean levels of LH and FSH were higher in the exposed group than those in the control group (P < 0.05), however, the mean levels of testosterone were lower in the exposed group compared to non-exposed ones (P < 0.05). A strong correlation was found between the concentration of lead fumes and the blood lead levels (r = 0.82; P = 0.003). Blood lead levels were inversely related to the testosterone levels and directly related to LH (r = 0.72; P = 0.004) and FSH (r = 0.78; P = 0.001) levels.

**Conclusions:**

Occupational exposure to metal fumes containing lead among welders may alter the level of sexual hormones and potentially harm the reproductive system.

## Background

Welding is a process in which two or more metals are bonded through pressure, heat, or both [1,2]. Shielded metal arc welding (SMAW) is a metal bonding process in which the arc between the electrode and the work piece is generated. The electrode coating provides a gaseous environment to prevent the air from entering the molten zone [3]. Millions of workers around the world are exposed to welding aerosols daily. This number even goes up to 730,000 in Europe [4]. Approximately 2 million full-time welders work in the world. There are 5.5 million welding-related businesses in Europe [5].

Fumes are very fine particles with a diameter of less than one micron which are produced by condensation of metal vapor during welding [6,7]. Fumes include a complex of different metal particles such as lead, ferrous, magnesium, zinc, beryllium, chromium, nickel, cobalt, cadmium, titanium, vanadium, antimony, and copper [8]. There have been increasing concerns about the harmful effects of welding fumes on the health of welders [9]. In fact, the metal fumes are absorbed through the lungs. Those are subsequently transported to different organs and affect their functions [10]. Welding fumes may cause variety of diseases such as asthma, bronchitis, lung fibrosis, cardiovascular complications, as well as central nervous and reproductive systems disorders [11,12,13,14,15]. Welding fumes have been classified as human carcinogens by the International Agency for Research on Cancer (IARC) and the European Union [16].

Inhalation of lead oxide fumes may cause lead poisoning [17]. Any amount of lead is considered to be harmful; although the American Conference of Governmental Industrial Hygienists (ACGIH) has recommended a contact limit of 0.05 mg/m^3^ for lead fumes [18]. Lead has been proven to have harmful effects on different organs [19,20,21,22]. Lead may cause adverse effects on the reproductive health among males [23,24,25]. This is because lead has cytotoxic effects on testicular tissues by reducing the levels of antioxidants and free radicals. In fact, lead damages both sertoli and leydig cells which presents with impaired spermatogenesis and testosterone productions, respectively [26,27,28,29]. A blood lead level of 60 μg/dl or higher can reduce sperm count as well as sperm motility and concentration, while it produces abnormal sperm morphology which all together eventually lead to reduced testicular mass and infertility [30,31].

Male reproductive hormones play a significant role in growth and development of sperm. The concentration of steroid hormones such as Luteinizing Hormone (LH), Follicle Stimulating Hormones (FSH), and Testosterone are considered as a sensitive indicator of reproductive system performance. LH regulates spermatogenesis by stimulating interstitial cells as well as producing testosterone; while FSH along with testosterone works on spermatogonial tubes and stimulates production of spermatozoa [32]. Lead reduces both the testosterone levels and the number of LH receptors on Leydig cells while it destructs the Sertoli cells. As such, it causes a negative feedback on the pituitary-gonadal axis and increases both LH and FSH levels in order to modulate the resulted damages to testis [29,33,34]. This study aimed to determine the concentration of lead fumes in the breathing zone and blood lead and LH, FSH, and testosterone levels. Our purpose was to assess the relationship between exposure to lead-containing welding fumes and the levels of reproductive hormones.

## Methods

### Study Design

This cross-sectional study was carried out based on the data derived from a water transfer company in Iran. After a preliminary review of the industry, SMAW was selected as the main source of releasing welding fume. SMAW welders were all eligible to enter the study. A number of 85 welders and 80 male individuals were selected as the exposure and control groups, respectively. Inclusion criteria consisted of being male, age between 20–50 years, married, non-drinker, non-smoker, no use of medications that likely affect the sexual derive, as well as no history of infertility, any major illnesses that affect sexual function, and no history of drug use. Participants were all required to sign a consent form. Those who failed to complete the questionnaire were withdrawn from the study. The individuals were allowed to leave the study at any time. Patients were assured their demographic information will be kept confidential.

### Air Sampling and Analysis

All air sampling and analysis of welding fumes were done according to the Method Number 7300 of the National Institute for Occupational and Safety (NIOSH) using Mixed Cellulose Ester (MCE) filter (25 mm, 0.8 μm, SKC Inc., USA), personal sampling pump (224-PCMTX8, SKC, Inc.) [35]. Samples were prepared and analyzed by inductively coupled plasma (ICP) (Varian, Liberty-RL model, Italy) with detection limit of 0.01 μg l^–1^.

### Blood Sampling and Analysis

Plastic tubes containing heparin were used to collect 8 cc of blood after 8 to 10 hours of fasting in order to measure the lead levels. Those were sent to a lab immediately. The blood lead levels were determined by an atomic absorption spectroscopy (Chemtech, UK) according to the method NIOSH 8003 [36]. The levels of LH, FSH, and testosterone were determined by chemiluminescence immunoassay (CLIA) which uses the MAGLUMI full-automatic chemiluminescence immunoassay analyzer designated by Snibe diagnostics (Shenzhen, China). The same technician conducted all measurements to avoid any predispositions.

### Statistical Analysis

SPSS software version 21 (SPSS Inc., Chicago, IL, USA) was used for statistical analysis. Data were analyzed using descriptive statistics, Student’s t-test and Spearman’s correlation test. P values of less than 0.05 were regarded as statistically significant. The Shapiro–Wilk test was used to test the normality of the data.

## Results

A total of 165 individuals, including 85 welders as an exposure group, and 80 administrative staff as non-exposed group participated in this study. According to Table 1, age ranged from 25 to 51 years with a mean and standard deviation of 38.17 and 12.44 years, respectively. The welders exposed to fumes for a length of 4.2 to 14 years with mean of 8.77 ± 3.61 years. According to Table 1, the concentration of lead fumes among welders averaged 0.57 ± 0.12 mg/m^3^. This was significantly higher than the threshold limit values-time weighted average (TLV-TWA) by the American Conference of Governmental Industrial Hygienists (ACGIH) (0.05 mg/m^3^) [18]. Likewise, the blood lead level averaged 460.28 ± 93.65 μg/L which was significantly higher than the biological exposure index (BEI) recommended level by ACGIH (200 μg/L).

**Table 1:** Background characteristics and exposure data in exposed and non-exposed groups.

	Exposed (n = 85)		Non-exposed (n = 80)		P-value*

	Mean ± SD	Range	Mean ± SD	Range	

Age (year)	38.17 ± 11.04	25–50	32.82 ± 9.66	22–43	0.026
History of Welding (year)	8.77 ± 3.61	4.2–14	ND**	ND	0.017
Lead fume (mg/m^3^)	0.57 ± 0.12	0.20–0.73	ND	ND	0.013
Blood Lead (μg/L)	460.28 ± 93.65	150.46–589.24	10.41 ± 3.76	3.31–12.09	0.010

* P < 0.05 was considered as statistically significant. ** ND: Not Detected.

Table 2 compares the levels of the sexual hormone between two groups. The average level of testosterone in the exposure group (2.02 ± 0.62 ng/ml) was significantly lower than that of the control group (7.26 ± 1.84 ng/ml) (P = 0.019). In addition, the mean level of FSH in the exposed group (9.34 ± 2.56 mIU/ml) was significantly higher than that of the control group (5.50 ± 2.19 mIU/ml)(P = 0.024). Likewise, the average level of LH in the exposed group (21.05 ± 6.39 mIU/ml) was significantly higher compared to the values of control group (12.01 ± 5.36 mIU/ml) (P = 0.013).

**Table 2:** Reproductive hormones levels in exposed and non-exposed groups.

	Exposed (n = 85)		Non-exposed (n = 80)		P value*

	Mean ± SD	Range	Mean ± SD	Range	

FSH (mIU/ml)	9.34 ± 2.56	3.17–21.57	5.50 ± 2.19	1.61–11.42	0.024
LH (mIU/ml)	21.05 ± 6.39	5.23–31.66	12.01 ± 5.36	3.33–24.76	0.013
Testosterone (ng/ml)	2.02 ± 0.62	1.9–5.81	7.26 ± 1.84	3.73–10.35	0.019

Table 3 shows the results of Spearman correlation test between the concentration of lead fume, blood level levels, as well as blood levels of LH, FSH, and testosterone. There was a strong correlation between the concentration of airborne lead and its level in the blood of welders (r = 0.823, p = 0.003). A paradoxical correlation was found between the blood lead and testosterone levels (r = –0.625, p = 0.002); however, blood lead levels was directly related to both LH (r = 0.724, p = 0.001) and FSH (r = 0.789, p = 0.001) levels.

**Table 3:** Correlation between blood lead level with lead fumes and reproductive hormones.

	Blood Lead (µg/L)	

	r	P value

Lead fumes (mg/m^3^)	0.823	0.001
FSH (mIU/ml)	0.789	0.001
LH (mIU/ml)	0.724	0.004
Testosterone (ng/ml)	–0.625	0.002

## Discussion

Our finding indicates the welders have relatively high blood levels of lead which may adversely affect the reproductive system function [37]. The results of the study shows the average concentration of lead fumes among welders is significantly higher than recommended levels by ACGIH. According to the findings, mean blood level of lead in welders was 460.28 ± 93.65 μg/l. This is significantly higher than the recommended level by ACGIH. Likewise, lead concentrated in welders’ breathing zone and blood was as high as 11.4 and 10 times the recommended levels, respectively. The congruence between air and blood lead levels might be due to lack of using respiratory protective equipment by most of the welders (87.75%).

The results of this study show a statistically significant relationship between the concentration of lead fumes in respiratory zone and the level of blood lead among welders. This is consistent with findings of the study done by Dehghan et al. [38] Previous studies have also brought up similar results with ours in respect to blood lead levels of welders [38,39]. Dorosti et al. was also able to show blood lead levels of welders were significantly higher than that of the control group [40].

Previous studies demonstrate exposure to lead has adversely affected the human reproductive system. Our study also shows the levels of sex hormones are significantly different between welders and control subjects. In fact, the average blood testosterone levels among welders are significantly lower than that of the control group. According to the results, there is a paradoxical correlation between the blood lead and testosterone levels among welders. This finding is consistent with the results of Wang et al. in which the concentrations of testosterone among workers exposed to lead in battery plant was significantly lower than that of the control group [41]. They also showed a reverse association between blood lead and testosterone levels among workers exposed to lead [41].

There are also significant differences in the levels of pituitary-ovaries axis hormones such as LH and FSH between welders who were exposed to lead fumes compared to the control group. According to the results, the concentration of LH and FSH hormones in lead-exposed welders was significantly higher than those of non-exposed group. As such, a direct significant correlation can be described between blood lead and both LH and FSH levels. Taher et al. (2017) reported significantly higher levels of LH and FSH among workers who were exposed to lead particles than that of the non-exposed group [42]. Likewise, Vivoli and colleagues reported a reverse correlation between blood lead level and both LH and FSH levels in men with blood lead levels of 9 μg/dl or higher [43].

There are many other studies which support relatively high levels of both LH and FSH among lead-exposed workers. In a study on lead melting workers, the amount of LH in the exposed group was significantly higher than non-exposed group [44]. Likewise, Kumar et al. reported lower levels of both LH and FSH among welders who were exposed to lead fumes with a concentration higher than recommended values by TLV-TWA. They also found welders who worked longer than 10 years in welding industries had higher levels of blood FSH than those who worked less than 10 years as a welder [45]. Similarly, Pant et al. was able to show a significant inverse correlation between blood lead levels (5.29–7.25 μg/dl) and both mobility and concentration of sperms among lead-exposed men [46]. Chowdhury et al. reported an increased risk of sperm depletion and reproductive system dysfunction in men who were constantly exposed to lead [37].

Histological studies of semen also support the findings. In fact, sperm analysis of exposed individuals demonstrated morphological damages to sperm as well as reduction in the levels of fructose and benzene dehydrogenase in semen [47]. Welders who were exposed to lead carried a relatively low sperm count and motility compared to the control group [39]. There are, however, studies that did not come up with these results. Seleven and colleagues reported no changes in sperm quality among workers who were exposed to lead [48]. Likewise, it has been reported there was no relationship between blood lead levels and variations in hormonal parameters [49]. The observed inconsistency might be due to the differences in lead levels in the breathing zone and blood of exposed individuals, exposure length of time, or the effect of confounding factors such as toxic gases, heat, and ultraviolet radiations during welding process, or even a combination of all.

This study includes some limitations as follows:

– The impossibility of collecting semen samples because of some cultural issues.
– Having small sample size due to lack of sufficient facilities and human resources for measurement of environmental and biological parameters.
– Not considering other environmental parameters like noise, vibration, electromagnetic field, other metal fume and toxic gases effecting on reproductive parameters of welders.

Yet, further studies are recommended to be performed on this issue in order to eliminate the aforementioned limitations.

## Conclusion

According to the results of the study, welders are exposed to relatively high levels of lead fumes at work. An increased blood lead level may reduce the blood testosterone and increase both LH and FSH levels among welders which may cause reproductive disorders. Therefore, occupational health professionals are required to apply the preventive measures such as installing local exhaust ventilation systems, limiting exposure time, biological monitoring and periodic screening health examination, and finally, providing appropriate respiratory protection devices for welders.
